# Baicalein Nanofiber Scaffold Containing Hyaluronic Acid and Polyvinyl Alcohol: Preparation and Evaluation

**DOI:** 10.3906/sag-2001-123

**Published:** 2020-06-23

**Authors:** Kamel BACHIMAM, Ezgi EMUL, Necdet SAGLAM, Feza KORKUSUZ

**Affiliations:** 1 Nanotechnology and Nanomedicine Department, Institute of Graduate School of Science and Engineering,Hacettepe University, Ankara Turkey; 2 Department of Sports Medicine, Faculty of Medicine, Hacettepe University, Ankara Turkey

**Keywords:** Baicalein, hyaluronic acid, nanofiber, electrospinning, bone cancer, tissue engineering

## Abstract

**Background/aim:**

Bone tumor is one of the major causes of tissue bone loss, particularly after performing surgical excision operation to bone lesion that needs to be replaced by biomaterials and ensure a complete filling of tissue-loss spaces. The purpose of our study was to produce a nanofiber-based bone graft scaffold to fill the gaps resulted from bone cancer treatment and also capable of carrying functional molecules that can play a major role in preventing further cancer growth at the targeted bone tissue.

**Materials and methods:**

Electrospinning method was used in order to produce nanofibers from different kinds of polymers; Hyaluronic acid (HA), Polyethylene oxide (PEO) and Polyvinyl alcohol (PVA) blended with different concentrations of herbal antibiotic and anti cancer flavonoid molecules called Baicalein (BE). The morphological and chemical structures of scaffold samples were studied using Scanning Electron Microscope (SEM), Fourier Transform Infrared-spectroscopy (FT-IR) and Surface-enhanced Raman spectroscopy (SERS) Analysis.

**Results:**

The results showed production of homogenous nanofibers-based scaffold (diameter between 80 nm and 470 nm) that contains the polymers used in the spinning process and the entrapped Baicalein molecules within the nanofiber structure.

**Conclusion:**

It was concluded that successful formation of bone tissue mimicking scaffold can be achieved by using Electrospinning method that produces nonwoven nanofibers and at the same time can hold functional anticancer agent such as Baicalein, which may allow using these types of scaffold in bone cancer treatment procedures.

## 1. Introduction 

Incidence of local recurrence of osteosarcoma and giant cell tumor of the bone after joint salvage surgery is 10%–15% and 0%–65% [1], respectively. Local recurrence is more common in regions where it is difficult to perform wide resection at the tumor margins, such as pelvic lesions [1].

Baicalein (BE) is an antibacterial [2], antiinflammatory [3] and anticancer [4] flavonoid that stimulates angiogenesis [5], bone formation [6,7] and metabolism [u2932], osteoblast proliferation [u2933]. A study [14] revealed that BE arrested the local invasion of osteosarcoma. We want to investigate if we could integrate and characterize a BE containing Hyaluronic Acid (HA)-Polyethylene Oxide (PEO)-Transforming Growth Factor beta-2 (TGF-β2)-Polyvinyl Alcohol (PVA) composite by electrospinning.

Electrospinning system consists of polymer solution that puts in a high electrical field. The high voltage of applied electrical field will play a role in deforming the spherical shape of solution droplet, which possesses resistance forces like surface tension and solution viscosity. This process gives the droplet a conical shape called Taylor cone. The competition between all mentioned forces will reach a level at which the droplet will break down and a charged jet will go out far away from the solution and settle on the collector surface as a fiber [15]. The advantages of electrospun nanofiber influenced number of researchers to use it in bone grafting synthesis like Yashimoto et al., Kim et al. and Emul et al. [16–18]. Loading nanofibers with active ingredients, like bone morphogenic protein (BMP-7) to improve osteoinductivity [9] and *cis*-diammine-di-iodoplatinum as anticancer agent [19], has also been established.

Objectives of this study were the production of the BE containing HA-PEO-TGF-β2-PVA using electrospinning and characterizing the composite by Scanning Electron Microscopy (SEM), Fourier Transform Infrared (FT-IR) and Surface-enhanced Raman spectroscopy’s that can be used for coating the distal ends of the limb salvage implants to decrease and/or prevent local recurrence in the future. 

## 2. Materials and methods D

### 2.1. Materials

Sodium Hyluronate 11,000,000 g/mol (Complejo Industrial Bioibérica, City ?, Italy) was used as a natural polymer for nanofiber production. Polyethyleneoxide 600,000 g/mol (Sigma-Aldrich Corporation, St. Louis, MO, USA) and polyvinyl alcohol 500,000 g/mol (Sigma-Aldrich Corporation, St. Louis, MO, USA) were synthetically polymers for nanofiber formation. Baicalein, 270.24 g/mol (Sigma-Aldrich Shanghai Trading Co. Ltd.., Shanghai China) was added to the electrospinning solution. Human recombinant TGF-β2 5UG was purchased from Sigma-Aldrich Corporation St. Louis, MO, USA. Deionized water was provided at Hacettepe University (Turkey). 

### 2.2. Preparation of polymer solution

Two different polymer solutionswere prepared separately in order to produce nanofibers via the electrospinning technique. The first solution contained HA with PEO and TGF-β 2. The other solution contained PVA and Baicalein.

The first solution was prepared by suspending 0.1% (w/v) of HA in 5ml deionized water, stirredor 20 min at 29 oC, then 10% (w/v) PEO was added and mixed gently for 20 min at 29 oC to ensure that all PEO molecules have completely dissolved in the turbid white color solution. After that, slow-speed stirring was kept for 24 h in room temperature. 40 ng/ml of TGF-β 2 was then added to the previous solution and stirred for 10 min at room temperature.

The other solution was preparedby adding 7% (w/v) PVA slowly to 5 ml cold deionized waterand kept for 24 h at room temperature, and then it was stirred for 4 h at about 65 oC. The same procedure was done to prepare another three 5 ml PVA solutions and concentrations of Baicalein [0.2%, 0.5%, 1%, 2% (w/v)] were added, respectively. The final four solutions were stirred for 6 h at about 73 oC. 

### 2.3. Electrospinning of nanofibers

Scaffolds containing two kinds of nanofibers were produced using electrospinning method [20]. Briefly, the two different polymer solutions were filled separately in different two plastic tubes, and high electrical field (12–15 kV) was applied with about 15 cm distance between tip of needle and collector. The process has been performed at a room temperature, in which both types of produced nanofiber were collected on the same collector to ensure the homogeneity of scaffold structure. All parameters used during nanofiber spinning were applied as they mentioned by Bachimam et al. [21].

### 2.4. Characteristics evaluation of nanofiber scaffold

The resulted nanofiber scaffolds, both those which contained different Baicalein concentrations, and the control scaffold sample (with no Baicalein), were all coated with gold and studied by Scanning Electron Microscope (SEM) (FEI Quanta 200F, FEI Company, Eindhoven, Netherlands). The chemical composition of scaffolds samples was assessed by Fourier Transform Infrared Spectroscopy (FT-IR) (Infrared spectra were taken by ATR-Nicolet iS10, Thermo Scientific Instruments LLC, Madison, WI, USA).The chemical infrastructure of scaffold samples was studied by Raman Microscopy System (Delta Nu Inc., Laramie, WY, USA) with laser source at 785 nm.

## 3. Results

### 3.1. Nanofiber scaffold samples

In our study, we used the electrospinning apparatus to produce a sheath like scaffold structure with yellowish white color, and we noticed that both yellow color and elastic properties were increased with the increasing of Baicalein concentration inside spun nanofiber.

### 3.2. Characterization of nanofiber-based scaffold samples

#### 3.2.1. Scanning Electron Microscope Analysis

**Figure 1:**
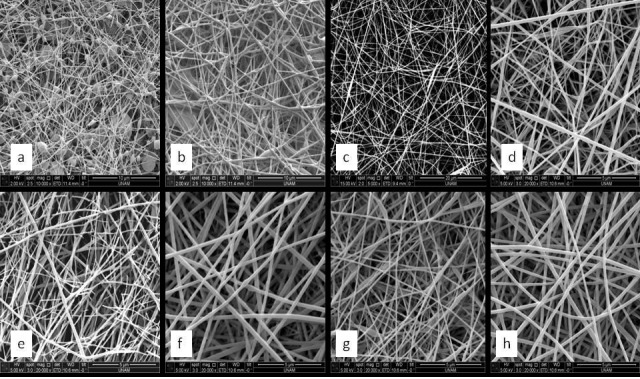
SEM images of our produced nanofibers containing; (a) HA/PEO 0.2% wt / 5% wt, (b) HA/PEO 0.1% wt / 10% wt, (c) PVA, (d) control sample (HA/PEO/TGF-β 2 + PVA) with 0% BE, (e) sample with 0.2% BE, (f) sample with 0.5% BE, (g) sample with 1% BE, and (h) sample with 2% BE.

The images that were taken for HA/PEO/TGF-β 2 [0.2% (w/v) / 5% (w/v) / 40 ng per ml] scaffold showed the presence of high beaded nanofibers (Figure 1a) that changed to be non beaded nanofiber with an average diameter at about 105 ± 20 nm (Figure 1b). Furthermore, we can notice (from Figure 1c) that PVA nanofibers were smooth nonwoven with average diameter at about 200 ± 22 nm. Our produced HA/PEO/TGF-β 2 and PVA/Baicalein nanofibers based scaffolds images (Figures 1d–1h) showed a smooth margins nonwoven and non beaded nanofiberscontaining different Baicalein concentrations (0%, 0.2%, 0.5% 1%, 2%) with average diameter at 175 nm, 120 nm, 270 nm, 137 nm, 280 nm, respectively. 

#### 3.2.2. Fourier transform infrared spectroscopy analysis 

**Figure 2:**
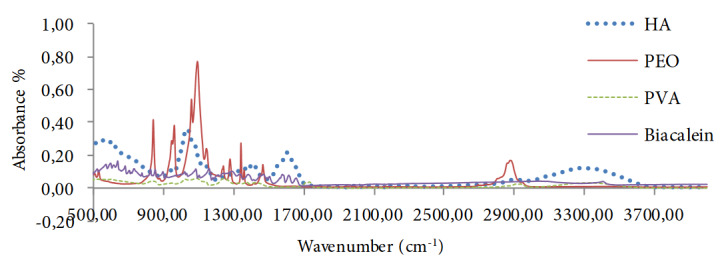
FT-IR spectra of the HA, the PEO, the PVA and BE, that shows the characteristic bands for each substance used in nanofiber formation and ensures the presence of these material in the end of spinning process.

FTIR analysis was performed for our produced nanofibers that showed the characteristic absorbance band of HA at 3291 cm-1, 1601 cm-1 and 1033 cm-1 that belonged to carbonyl (CO), hydroxyl (OH) and amine (NH) moieties, respectively (Figure 2) [22]. Characteristic bands for PEO were also seen clearly at 2889 cm-1 and 1090 cm-1, which attributed to the (CH) and (COC) groups, respectively (Figure2) [23]. PVA absorbance bands were noticed at 3302 cm-1, 2901 cm-1,1403 cm-1and 1087 cm-1 that correlate with (OH), (CH), (CHOH) and (CO) groups, respectively [24].

Absorbance bands for Baicalein were also observed at 3400 cm-1, 1655 cm-1,1614 cm-1 ,1470 cm-1, 1292 cm-1, 1159 cm-1, 1083 cm-1 , 880 cm-1, 825 cm-1, 637 cm-1 and 467 cm-1, which contributedto (OH) stretching, (CC) stretching, (CCO) bending, (HCC) bending (from 1292 to 1083 cm-1) , (CCO), (HCCC) torsion and (CCC) bending (637 to 426 cm-1), respectively (Figure 2) [25]. 

**Figure 3:**
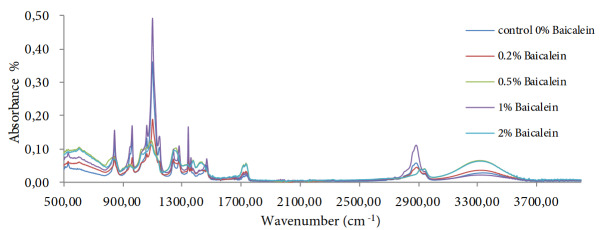
FT-IR spectra of the produced scaffolds with different concentration of Baicalein. Comparing to the spectra of control scaffold containing HA/PVA/TGF β 2, the absorption of BE seen to be increased to the increasing of the loaded Baicalein’s concentrations 0.2%, 0.5%, 1% and 2%, respectively.

In the same way, we assessed the chemical composition of our produced PEO/HA/TGF-β 2 with PVA/Baicalein nanofibers scaffold by FTIR analysis method. As noticed in Figure 3, the characteristic absorbance peaks for HA [at 3328 cm-1, at 1600 cm-1 and at 1044 cm-1 correlating with (OH), (CO), and (NH) groups, respectively] , PEO [at 2880 cm-1 for (CH) stretching and 1100 cm-1for (COC) group] and PVA [at 3312 cm-1, 2940 cm-1, 1428 cm-1 and 1089 cm-1correlating with (OH), (CH), (CHOH) and (CO) respectively]. Furthermore, in all scaffolds samples we can notice that two characteristic absorbance bands for BE (at 3300 cm-1 and 630 cm-1) were increased gradually correlating with the increasing of BE concentration inside scaffolds structures, as seen in Figure 3. 

#### 3.2.3. Surface-enhanced Raman spectroscopy (SERS) analysis 

**Figure 4:**
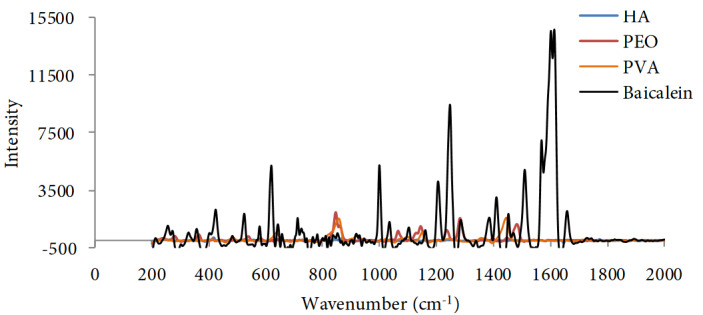
Raman spectra of the HA, the PEO, the PVA and BE; this diagram ensures the formation of spun-nanofiber without loss of any of the used materials.

**Figure 5:**
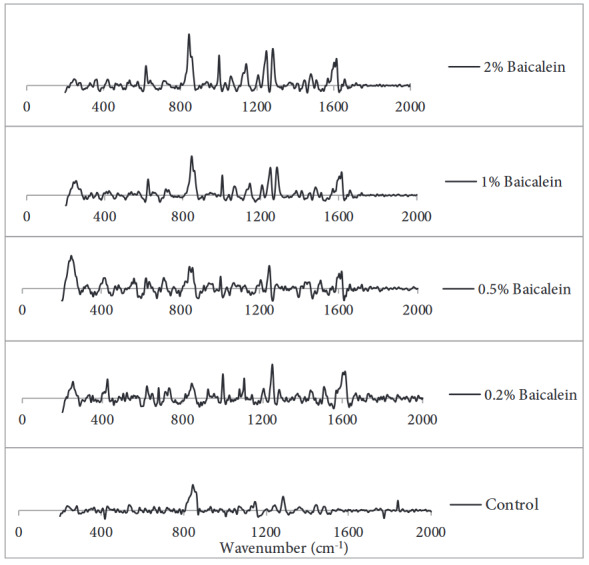
Raman spectra of the control scaffold containing (HA/PVA) and the other scaffolds loaded with different BE concentrations. It shows the gradual increasing in band intensity corresponding to the increasing in BE concentration of spun-nanofibers especially at 1290 cm-1, 1005 cm-1 and 670 cm-1 .

We used SERS for materials analysis. As it can be seen in Figure 4, the spectrum of HA exhibit a sharp band at 1047 cm-1, 1328 cm-1, 1378 cm-1 and 1662 cm-1 wavenumbers correlating with (C-C and C-O stretching), Amid III, C-H and C=C bonds, respectively. PEO spectra shows characteristic peaks at 1480 cm-1, 1281 cm-1, 1140 cm-1 and 840 cm-1 corresponding with CH2 scissoring, C-O-C, CC-COC stretching and CH2 waging respectively. In the same way, we can notice the PVA spectra in which the peaks at 1360 cm-1 and 1440 cm-1correlating with C-H and O-H bending respectively. BE shows sharp bands at 1620 cm-1, 1450 cm-1, 1290 cm-1, 1005 cm-1 and 670 cm-1 correlating with C=O stretching, CC stretching, CO stretching , HCC and CCC bonding, respectively [25,26]. On the other hand, from Figure 5 we can notice the Raman spectrum of our produced scaffold samples in which the characteristic peaks of BE were not seen in control spectrum, while we can easily notice the peaks of BE absorbance band of at 1640 cm-1, 1290 cm-1, 1005 cm-1 and 670 cm-1 and how they increased according with BE concentration inside the scaffold structure.

## 4. Discussion 

### 4.1. Polymer Solution Preparation

We faced many problems during preparation of polymer solutions. By keeping the solution stirring for 24 h, water evaporation process had affected the initial concentrations of dissolved materials. To solve this problem, the amount of evaporated water during 24 h was calculated and replaced at the end of stirring.

On the other hand, while we were adding Baicalein to PEO/HA solution and mixed them together to produce HA/PEO/Baicalein nanofiber, Baicaleinwas added to PEO solution andheated, but after 15 min, black particles started to appear inside the solution and gradual change of solution’s color from yellow to green then to black was clearly observed. We repeated the trial three times with presences of HA and other trials without adding HA and we got the same result. This phenomenon maybe due to an oxidation reaction between Baicalein and PEO solution, and that made PVA a good replacement to PEO to be mixed with BE in order to avoid oxidation.

### 4.2. Electrospinning and Nanofiber Production 

Electrospinning method has attracted the attention because of its great ability to produce synthetic scaffold structure with high degree of bone tissue similarities that comes from its contents of fibers with a diameter varying from 70 nm till 500 nm, and also relatively big pours (5–6 µm) which plays a big role in cell movement and migration as well as blood vessels formation and angiogenesis [u2987]. This property greatly depended on the homogeneity of the generated scaffold nanofibers that was considered seriously during our work by spinning 0.4 mL from one solution, then spinning 0.4 mL from the other polymer solution. This process was repeated until 2 mL of each solution has been spun.

Although this technique seemed to be a good step to ensure a high degree of homogeneity between the spun nanofibers inside the scaffolds samples, it affected also the quality of SEM imaging, FT-IR and Raman analysis, because the upper layer of the resulted scaffold may contain substances of just one solution, which may give incomplete information during nanofiber characterization tests. To overcome this problem, the newly formed nanofiber layers were mixed multiple times to ensure presence of all materials on the surface of our scaffolds. Eventually, samples for upcoming tests were taken from different places of scaffolds surfaces.

### 4.3. Assessment of nanofiber characteristics 

#### 4.3.1. SEM images

SEM images showed the strong relation between the concentration of materials used in nanofiber process and the shape, properties and average diameter of resulted nanofibers. Even presence of beads within the spun fibers was also affected by the amount of materials used in the work stages. Figure 1a demonstrated the final results of 0.2% (wt) / 5% (wt) HA/PEO polymer solution electrospinning, which clearly showed the huge amount of beads formed inside the scaffold structure, compared with complete beads disappearance when spinning 0.1% (wt) / 10% (wt) HA/PEO solution (Figure 1b).

Furthermore, nanofibers average diameter changed respectively with the increasing in BE concentration during scaffold production. The smallest average diameter was at about 80 nm in control samples (without BE), and then started to increase till it reached 470 nm in 2% BE scaffold. These results could be due to the increasing in the solution viscosity correlating with the increasing in amount of material spun out of solution and then rearranged to be fibers. 

#### 4.3.2. FT-IR analysis

A general observation from the FT-IR analysis test results revealed that scaffolds samples absorption spectra possesses number of peaks, particularly at 3320 cm-1 and at 620 cm-1 correlating with both OH stretching and CCC bending, respectively. The peaks of those two bands significantly increased corresponding with rising up in BE concentration (Figure 3). Other characteristic of BE bands that expected to be seen in the IR results were disappeared or being very small, that can be as a result of the masking effect from other materials to number of BE absorption bands.

#### 4.3.3. Surface-enhanced Raman spectroscopy analysis 

SERS analysis was performed to obtain information on the chemical structure of the resulted scaffolds and detect any changes in material concentration. There are number of previous studies had detected the interaction of BE particles with other spun elements by getting benefit of the high analytical capability of SERS, such as studying the characteristics of PVA/Baicalein spun nanofibers by Kim et al. [29]. In our study, we detected the chemical characterizations of the novel composition (HA/PVA/BE) of our resulted scaffolds. From Figure 4, we can clearly notice that the characteristic bands of Baicalein, which were not found in the control sample spectra, started to appear and increased respectively with the rising amount of BE concentrations, particularly at 1620 cm1, 1450 cm-1, 1290 cm-1, 1005 cm-1 and 670 cm-1 (Figure 5). The spectra also gave a clue that no new chemical bonds were formed, but it still need to be detected by further tests especially in case of conducting in vivo studies or even in clinical application trials.

### 4.4. Limitations and forward applications

We discussed some difficulties that we faced during our work and how we found solution for it, however we still notice some limitations that we should mention in the scope of our work discussion. First, we do not form or test more scaffold samples containing higher BE concentrations that might give an important clue on the effect on both low and high BE concentrations on the formed nanofibers nature and also the integration with other scaffold elements. Second, due to lack of time, we did not perform any in vitro or in vivo studies to evaluate the effects of scaffolds on both normal cells and tumor cell, to estimate the level of scaffold integration and bioavailability within the bone tissue and to measure the quality formation of new fresh tissue in place of degraded transplanted scaffold, that might have a big role in further research planning and forward surgical and medical utilizations.

However, our results seemed to have many promising applications. According to recent studies, there are various evidences on the effectiveness of BE against some types of cancer cells that had gained chemotherapy and radiotherapy resistance [30] and high invasion ability tumor [31], particularly osteosarcoma bone tumor [32] that, in some cases, need to be excised and refilling the loss of bone tissue with suitable synthesized graft. Scientists can take advantage of the promising antioncogenic property of BE with other antiinflammatory and antibacterial features by getting it carried and released in a sustained pattern from nanofibers-based scuffled graft, which would ensure significant reconstructional in segmental bone loss by remodeling and regeneration processes [33–35], and in the same time, preventing any probable infectious complications or tumor recurrence in the newly formed tissue and reducing the need of higher dose of chemotherapy by controlling the local environment [36]. 

In conclusion, we successfully produced scaffold-based nanofibers that mimicking the normal bone tissue by applying the electrospinning method on two polymer solutions (HA/PEO/TGF-β 2 and PVA nanofiber scaffolds containing different concentration of Baicalein) with a nanofiber diameter ranging from 80 nm to 470 nm. We furthermore assessed the morphology and characterization of our produced nanofiber by studying their SEM images and also the analysis results of FT-IR spectroscope and Raman spectroscope that gave us a clear idea about the chemical composition of the produced scaffold samples. Baicalein loaded nanofibers have a wide range of applicability as a biomedical material that can be used for prevention of local recurrence of bone tumors and ensuring effective tissue remodeling and regeneration.

## Acknowledgments

We wish to thank Prof. Dr. Celal ÜLGER (Department of Molecular Biology, Biology Department, Faculty of Arts and Sciences, Aydın Adnan Menderes University, Aydın, Turkey) for supplying the Baicalein. Ass. Prof. Memed DUMAN and his staff from Nanotechnology and Nanomedicine Division, Institute of Science (Department of Basic Pharmaceutical Sciences Collaborations, Faculty of Pharmacy) Hacettepe University, Ankara, Turkey allocated their laboratory infrastructure for the experiments. 

## Disclaimers/Conflict of interest

The Scientific Research Projects Coordination Unit at Hacettepe University, Ankara, Turkey (Project Number: 9112) funded this study. This research was part of the MSc. thesis of Kamel BACHIMAM. The authors declare no conflict of interest.

This study was presented at the Taiwan-Turkey Science Summit entitled “Translation of Cells, Nanomaterials and Signaling Molecules into Regenerative Medicine” between April 1 to 3, 2018.
